# *Streptococcus thoraltensis* Bacteremia: First Described Case as a Fever of Unknown Origin in Human

**DOI:** 10.1155/2018/7956890

**Published:** 2018-12-02

**Authors:** Nikolaos Petridis, Athanasia Apsemidou, Georgios Kalopitas, Georgios Pilianidis, Iakovos Avramidis

**Affiliations:** First Department of Internal Medicine, G. Papanikolaou General Hospital, Thessaloniki, Greece

## Abstract

*Streptococcus thoraltensis* has mainly been reported to cause infections in animals. Its clinical significance as a human pathogen has not yet been fully elucidated and needs further investigation. We describe here a case of bacteremia attributed to *S. thoraltensis* in a 55-year-old female patient admitted to our department due to fever of unknown origin. To the best of our knowledge, this is the first reported case of *S. thoraltensis* bacteremia in a human and the first reported case of *S. thoraltensis* as a cause of fever of unknown origin in human.

## 1. Introduction

*Streptococcus thoraltensis* is a recently described species that has been isolated from several animal species [1]. According to the literature, this rare and unusual species of streptococci has recently been isolated from human samples. A recent study revealed *S. thoraltensis* as a predominant colonizing isolate in the nasal cavity and oropharynx in 29 fuel workers [2]. To the best of our knowledge, only one case of *S. thoraltensis* isolation from both the maternal placenta and newborn tracheal aspirate cultures and four cases from the subgingival plaque in patients undergoing tooth extraction have been reported in the literature [3–5].

## 2. Case Presentation

A 55-year-old female patient with past medical history of osteoporosis and hyperlipidemia was admitted to our department for a 2-month history of high-grade fever, up to 39°C, mainly occurring in the evening, which was responding to acetaminophen. She denied night sweats, anorexia, fatigue, weight loss, chest pain, shortness of breath, other joint pain, abdominal pain, recent travel, or a family history of autoimmune disease, although she reported traumatic dental procedure 6 months prior to admission.

On presentation, her vital signs revealed a temperature of 36.7°C, heart rate of 74 bpm, and blood pressure of 100/70 mmHg. Her physical examination was unremarkable, except for a systolic heart murmur, heard best over the aortic, pulmonic, and mitral area with radiation into the neck. Laboratory studies revealed thrombocytopenia with 123.000 *μ*L platelets, white blood cell count of 4.3 × 10^9^ L with neutrophils as the predominant type, and mild normocytic anemia (Hct: 34.7%; Hb: 11.7 g/d). The C-reactive protein (CRP) was 1.43 mg/dL (nl: <0.5), the erythrocyte sedimentation rate (ESR) was 32 mm/h, and creatine phosphokinase (CPK) was also elevated as 335 U/L (reference range: <180 U/L).

From three blood cultures obtained on the 1st, 2nd, and 3rd day of her hospitalization, when she was febrile, *Streptococcus thoraltensis* was identified, while infection caused by Brucella, *Mycobacterium tuberculosis*, Coxiella, and Leishmania was excluded. The suspicion of an infective endocarditis (IE) was raised, and a transthoracic and transesophageal echo was performed. However, there was no evidence of IE, and only mild aortic stenosis was revealed. A panoramic X-ray showed no lesions. Additionally, as part of the diagnostic procedure, a chest, abdomen, and pelvis computed tomography (CT) scan was performed and revealed no abscesses and masses.

**Figure 1:**
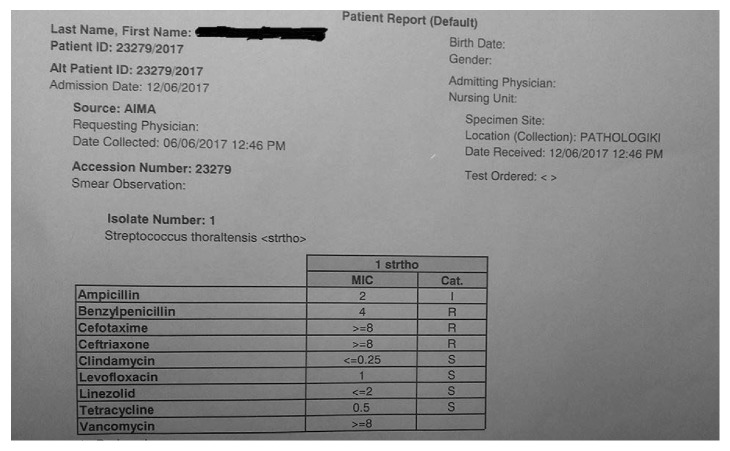
Susceptibility test of *Streptococcus thoraltensis* isolate.

Due to the fact that the patient continued to have high-grade fevers and the systolic heart murmur, empirical antibiotic treatment with ampicillin/sulbactam and gentamicin was initiated on day 4. Susceptibility testing was performed on the initial isolate and revealed intermediate sensitivity to these agents, as determined by the E-test method (Figure 1). The patient responded 48 hours after the initiation of the treatment. The duration of the antibiotic therapy was fourteen days. The patient had experienced no recurrence of fever, the subsequent blood cultures were negative, and the inflammation markers decreased (CRP: 0.3 mg/dl; ESR: 10 mm/h).

## 3. Discussion

We searched the literature including PubMed and individual references for publications of review articles, single cases, or case series with the following keywords: “Streptococcus thoraltensis” and “human.” There have been documented only five cases of *S. thoraltensis* isolation from human samples. To the best of our knowledge, this is the first reported case of *S. thoraltensis* bacteremia.

A significant limitation to this report is the method used to identify the pathogen due to the fact that the automated VITEK 2 system lacks the specificity of the 16S rRNA gene sequence, although a proper gold standard for the identification of streptococci is not available [6–8]. Nevertheless, identification of *S. thoraltensis* from three individual cultures presents a strong case to implicate this organism.

The characteristic of our patient's infection was high fever mainly occurring in the evening and relatively low levels of inflammation markers which nevertheless were decreased after the treatment initiation. Although our patient had a good clinical outcome, the case was clinically challenging regarding the lack of *S. thoraltensis* cases and the unknown proper duration of treatment in this isolate's bacteremia. Regarding the source of the infection, it remains unknown, considering the long period between the dental procedure and the initiation of her symptoms (>4 months). Hence, her bacteremia can be considered community acquired of unknown origin.

*S. thoraltensis* has mainly been reported to cause infections in animals. Its clinical significance as a human pathogen has not yet been fully elucidated and needs further investigation. Whether this case represents an isolated and rare bacteremia or an emerging infection will be established by novel possible cases.
